# Targeting of tolerogenic dendritic cells to heat-shock proteins in inflammatory arthritis

**DOI:** 10.1186/s12967-019-2128-4

**Published:** 2019-11-14

**Authors:** Rachel Spiering, Manon A. A. Jansen, Matthew J. Wood, Anshorulloh A. Fath, Oliver Eltherington, Amy E. Anderson, Arthur G. Pratt, Willem van Eden, John D. Isaacs, Femke Broere, Catharien M. U. Hilkens

**Affiliations:** 1 Translational and Clinical Research Institute, Faculty of Medical Sciences, Newcastle University, Newcastle upon Tyne, UK; 2 Research into Inflammatory Arthritis Centre Versus Arthritis, (Formerly: Arthritis Research UK Rheumatoid Arthritis Pathogenesis Centre of Excellence (RACE)), Newcastle upon Tyne, UK; 3 NIHR-Newcastle Biomedical Research Centre in Ageing and Long-Term Conditions, Newcastle Upon Tyne Hospitals NHS Foundation Trust and Newcastle University, Newcastle upon Tyne, UK; 4 Division of Immunology, Department of Infectious Diseases and Immunology, Utrecht University, Utrecht, The Netherlands; 5 Department of Clinical Sciences of Companion Animals, Faculty Veterinary Medicine, Utrecht University, Utrecht, The Netherlands

**Keywords:** Human, Inflammatory arthritis, Heat shock proteins, Tolerogenic dendritic cells, Tr1

## Abstract

**Background:**

Autologous tolerogenic dendritic cells (tolDC) are a promising therapeutic strategy for inflammatory arthritis (IA) as they can regulate autoantigen-specific T cell responses. Here, we investigated two outstanding priorities for clinical development: (i) the suitability of using heat-shock proteins (HSP), abundant in inflamed synovia, as surrogate autoantigens to be presented by tolDC and (ii) identification of functional biomarkers that confirm tolDC regulatory activity.

**Methods:**

Cell proliferation dye-labelled human peripheral blood mononuclear cells of IA (rheumatoid arthritis (RA) and psoriatic arthritis (PsA)) patients or healthy donors were cultured with HSP40-, HSP60- and HSP70-derived peptides or recall antigens (e.g. tuberculin purified protein derivative (PPD)) in the presence or absence of tolDC or control DC for 9 days. Functional characteristics of proliferated antigen-specific T-cells were measured using flow cytometry, gene expression profiling and cytokine secretion immunoassays. Repeated measures analysis of variance (ANOVA) with Bonferroni correction for comparisons between multiple groups and paired Student *t* test for comparisons between two groups were used to determine significance.

**Results:**

All groups showed robust CD4^+^ T-cell responses towards one or more HSP-derived peptide(s) as assessed by a stimulation index > 2 (healthy donors: 78%, RA: 73%, PsA: 90%) and production of the cytokines IFNγ, IL-17A and GM-CSF. Addition of tolDC but not control DC induced a type 1 regulatory (Tr1) phenotype in the antigen-specific CD4^+^ T-cell population, as identified by high expression of LAG3, CD49b and secretion of IL-10. Furthermore, tolDC inhibited bystander natural killer (NK) cell activation in a TGFβ dependent manner.

**Conclusions:**

HSP-specific CD4^+^ T-cells are detectable in the majority of RA and PsA patients and can be converted into Tr1 cells by tolDC. HSP-loaded tolDC may therefore be suitable for directing T regulatory responses to antigens in inflamed synovia of IA patients. Tr1 markers LAG3, CD49b and IL-10 are suitable biomarkers for future tolDC clinical trials.

## Background

Rheumatoid arthritis (RA) arises from a breakdown in self-tolerance leading to aberrant immune responses to autoantigens. Current treatments involve chronic immunosuppression in a non-antigen specific manner. Although these treatments can be effective at alleviating symptoms they do not provide a cure and the associated general immunosuppression can cause unwanted side effects (e.g. increased susceptibility to infection and certain cancers). An alternative approach are treatments that reinstate self-tolerance, leading to long-term remission whilst leaving protective immunity intact.

An emerging tolerogenic strategy is the administration of tolerogenic dendritic cells (tolDC). These cells act by inhibiting T-cell mediated pathology, for example through the induction of regulatory T-cells (Treg) [1, 2]. We recently conducted a clinical trial of autologous tolDC treatment in both RA and psoriatic arthritis (PsA) patients, confirming the safety and feasibility of this approach [3]. However, two critical and related issues were highlighted. The first relates to identification of the optimal target (auto)antigen(s). Because definitive arthritogenic autoantigens have not been identified, for our clinical trial we pragmatically ‘loaded’ tolDC with autologous synovial fluid, based on data suggesting a content of relevant patient-specific autoantigens [4]. However, without knowledge of the targeted antigen(s)’ identity it was not possible to measure modulation of the antigen-specific T-cell response. The second issue is the lack of suitable biomarkers. Because tolDC act in a highly targeted manner, it is imperative to monitor changes in antigen-specific T-cells, rather than measuring systemic immune markers. Loading of tolDC with known antigens will enable immune monitoring in a highly specific manner. Thus, future therapeutic studies with tolDC can be greatly improved by loading tolDC with relevant and known antigens, facilitating immune monitoring at the antigen-specific level and defining biomarkers of tolDC effectiveness.

We recently suggested to load tolDC with the surrogate self-antigens heat-shock proteins (HSPs) [5]. HSPs are molecular chaperone proteins highly expressed in inflamed tissue. Indeed, the expression of HSP40, HSP60 and HSP70 family members is upregulated in the synovial tissue of RA patients [6–9] and in the inflamed tissues of patients with other autoimmune diseases like multiple sclerosis, atherosclerosis, juvenile dermatomyositis and juvenile idiopathic arthritis [10–14]. Moreover, adoptive transfer of HSP-specific Treg effectively suppressed established disease in a murine autoimmune arthritis model. Subsequent deletion of these donor HSP-specific Tregs completely reversed the inhibition of disease progression, indicating disease suppression was induced by HSP-specific Tregs and not via bystander suppression [15]. Thus, it is likely that directing a regulatory T-cell response to a non-disease inducing antigen present in the diseased tissue is sufficient to dampen down pathogenic autoimmune responses.

To this aim, we (1) assessed the presence and phenotype of HSP-specific T-cells in RA and PsA patients and healthy donors; (2) investigated the ability of tolDC to induce a regulatory phenotype in HSP-specific T-cells, and (3) identified suitable biomarkers for the identification of tolDC-modulated T-cells that can be used for imminent clinical trials.

## Methods

The minimum information about tolerogenic antigen presenting cells (MITAP) checklist was followed for the preparation of this paper [16].

### Peptides and antigens

HSP40 peptide: DnaJP1: QKRAAYDQYGHAAFE, HSP60 peptides: p1: GEALSTLVVNKIRGT and p3: PYILLVSSKVSTVKD, HSP70 peptide: B29: VLRIVNEPTAAALAY and a negative control peptide A5: RQAILTLQTSSSEPR (Genscript). Whole antigens that were used were tuberculin purified protein derivative (PPD; Statens Serum Institut) and *Candida albicans* (CA; Soluprick; Alk).

### Isolation of cells

Human blood samples were obtained from healthy controls (HC) and treatment-naïve patients with recent onset arthritis (PsA and RA). Samples were collected with informed consent and following a favourable ethical opinion from local ethics committees. Peripheral blood mononuclear cells (PBMC; from 40 ml EDTA blood per donor) were isolated as previously described [17]. Monocytes were positively selected from PBMC using anti-CD14 microbeads (Miltenyi Biotec) according to manufacturer’s protocol with one minor change: 10 µl instead of 20 µl anti-CD14 beads per 1 × 10^7^ cells was used for cell isolation. CD14-depleted PBMC (hereafter referred to as ‘PBMC’) were collected from the column flow-through and stored for 1 week at − 80 °C in FCS (Gibco) with 10% DMSO (Sigma) and were used for the measurement of HSP-specific T cell responses and the DC/PBMC co-culture experiments (see below).

### Establishment of tolDC

Immediately after isolation, monocytes were cultured in 24 wells plates (Corning) at 0.5 × 10^6^ cells/ml (total 1 ml/well) for 7 days in CellGenix DC medium (CellGenix) containing penicillin (100 U/ml), streptomycin (100 μg/ml), GM-CSF (50 ng/ml; Immunotools) and IL-4 (50 ng/ml; Immunotools). During this period cells were kept at 37 °C with 5% CO_2_. On day 3, half of the medium was substituted by fresh (warm) medium containing GM-CSF (100 ng/ml) and IL-4 (100 ng/ml). For the generation of tolDC, dexamethasone (1 μM; Sigma) was added on days 3 and 6 and 1,25-dihydroxyvitamin D3 (Calcitriol; 0.1 nM; Tocris) and monophosphoryllipid A (MPLA) (1.0 μg/ml; Invivogen) were added only on day 6. Immature DC (imDC) were cultured in the presence of GM-CSF (50 ng/ml) and IL-4 (50 ng/ml). On day 7, 24 h after the last treatment, DC were harvested and washed extensively before functional assays were performed. DC were then resuspended at 4 × 10^5^ cells/ml in X-VIVO-15. DC phenotype was checked using flow cytometry and was consistent with tolDC exhibiting a semi-mature phenotype, expressing low levels of CD83, intermediate levels of CD86 and high levels of HLA-DR and TLR2 (data not shown).

### Measurement of HSP-specific T cell responses

PBMC were thawed, washed and labelled with 0.2 μM carboxyfluorescein succinimidyl ester (CFSE; eBioscience) or 0.2 μM cell proliferation dye eFluor-450 (CTV; eBioscience) in PBS for 10 min at 37 °C. CFSE/CTV was quenched with 10% human serum (HS; Sigma) in HBSS (Lonza). Cells were resuspended at 2 × 10^6^ cells/ml in X-VIVO-15 medium (Lonza) supplemented with 4% HS (final concentration 2%) and plated at 2 × 10^5^ cells per well (96 wells; round bottom; Corning). For each peptide eight wells were prepared. Peptides were added at 10 µg/ml. Cells were cultured for 9 days at 37 °C with 5% CO_2_. At the end of the culture, supernatants were collected for cytokine determination. Depletion of CD14 from PBMC did not hamper detection of HSP-specific T cell responses (data not shown).

### DC/PBMC co-cultures

CFSE or CTV-labelled PBMC were resuspended at 4 × 10^6^ cells/ml in X-VIVO-15 medium (Lonza) supplemented with 8% HS (final concentration 2%) and plated at 2 × 10^5^ cells per well (96 wells; round bottom; Corning). tolDC or, as a control, imDC were added in a 1:10 ratio (i.e. 2 × 10^4^/well), in the absence or presence of PPD (1 µg/ml) CA (1 µg/ml) or a cocktail of the HSP-peptides (HSP40 DnaJP; HSP60 p1 and p3; HSP70 B2; all at 4 µg/ml) Cells were cultured for 6 (IL-10 secretion) or 9 days (all other measurements) at 37 °C with 5% CO_2_. TGF-βRI (ALK5) inhibitor (SB-505124; 1 µM; Sigma) was added where indicated. At the end of the culture, supernatants were collected for cytokine determination.

### Flow cytometry

For cell surface staining: cells were washed in flow cytometry buffer (PBS (Lonza) supplemented with 3% fetal calf serum (FCS; Gibco), 1 mM EDTA (Fisher Scientific) and 0.01% sodium azide (Sigma)) before incubating them for 30 min on ice in flow cytometry buffer containing antibodies and 4 µg/ml human immunoglobulin (Ig)G (Grifols). Cells were washed and resuspended in flow cytometry buffer before analysis. For intracellular cytokine staining (ICS): cells were first stimulated with PMA (50 ng/ml) and ionomycin (1 μg/ml) for 5 h in the presence of brefeldin A (1 μg/ml; all from Sigma Aldrich) at 37 °C, 5% CO_2_. Cells were then surface stained as described before, washed in flow cytometry buffer and fixed/permeabilised using cytofix/cytoperm buffer (BD Biosciences) for 30 min on ice. Cells were washed twice in 1× perm wash buffer (BD Biosciences) and stained for 30 min in 1× perm wash buffer containing antibodies and 8% mouse serum. Cells were washed once in 1× perm wash buffer and once in flow cytometry buffer before resuspending them in flow cytometry buffer. Data were collected on an LSRfortessa X20 (BD Biosciences) and analysed using FlowJo (Tree Star Inc). Additional file 1: Table S1 depicts antibodies and live/dead dyes used for analysis.

### RNA isolation and gene expression analysis

Cells from the PPD-DC/PBMC co-cultures were harvested and a total of 100,000 CFSE^−^CD4^+^DAPI^−^ cells per sample were sorted into RLT buffer (Qiagen) supplemented with 1% β-mercaptoethanol (Sigma) using a FACSAria-fusion sorter (BD Biosciences). Lysates were stored for up to 4 months at − 80 °C before isolation of RNA. RNA was isolated using an RNeasy Micro Kit (Qiagen; including DNase step) according to the manufacturer’s protocol. A total of 100 ng of RNA was used for gene expression profiling by the nCounter technology platform (NanoString; Human Immunology Panel), performed according to the manufacturer’s protocol. Data was analysed using nSolver™ Analysis Software version 4.0 and R.

### IL-10 secretion

Cells from PPD-DC/PBMC co-cultures were harvested on day 6 and a minimum of 25,000 CFSE^−^CD4^+^ (unfractionated proliferated CD4^+^ T-cells from control-PPD cultures) or CFSE^−^CD4^+^LAG3^+^CD49b^+^ (proliferated Tr1 cells from tolDC-PPD cultures) sorted into X-VIVO-15 with 20% HS, using a FACSAria-fusion sorter. Sorted cells were rested for 2 days in X-VIVO-15 supplemented with 2% HS and 10 IU/ml of IL-2 (Proleukin; 25,000 cells/well; 96-well round-bottom). Cells were subsequently washed and restimulated with 10 µg/ml platebound anti-CD3 (OKT3; Biolegend) and 1 µg/ml soluble anti-CD28 (CD28.2; Biolegend) in X-VIVO-15 supplemented with 2% HS (total 100 µl; 96-well flat-bottom). Supernatants were collected after 72 h for cytokine determination.

### Cytokine secretion

Cytokine production was determined in supernatants by Meso Scale Discovery (MSD; U-Plex (IL-10, IFNγ, IL-4, IL-17A, GM-CSF) or by sandwich ELISA from BD (IL-10).

### Statistical analysis

The following statistical analyses were performed using Prism 5: repeated measures analysis of variance (ANOVA) with Bonferroni correction for comparisons between multiple groups, paired Student t-test for comparisons between two groups.

## Results

### Pro-inflammatory HSP-specific T-cells are present in IA patients

Our initial studies investigated whether immunodominant pan-DR-binding peptides from bacterial HSP40 (dnaJP1), mycobacterial (myc)-HSP60 (p1 and p3) and myc-HSP70 (B29) [14, 15, 18] could be recognised by peripheral blood CD4^+^ T-cells of healthy donors and IA patients (Table 1). We included both RA and PsA patients, because patients from both these IA groups participated in the phase I tolDC safety trial [3].

**Table 1:** Characteristics of RA and PsA patients and healthy donors

	RA (n = 19)	PsA (n = 10)	Healthy (n = 19)
Age, years	66 (60–70)	50 (34–56)	45 (29–52)
Sex, % females	68	50	63
Duration of symptoms, weeks	12 (7–40)	17 (8–37)	–
CRP, g/l	27 (9–53)	6 (5–19)	–

Except where indicated otherwise, values are the median (interquartile range)

*CRP* C-reactive protein

**Fig. 1:**
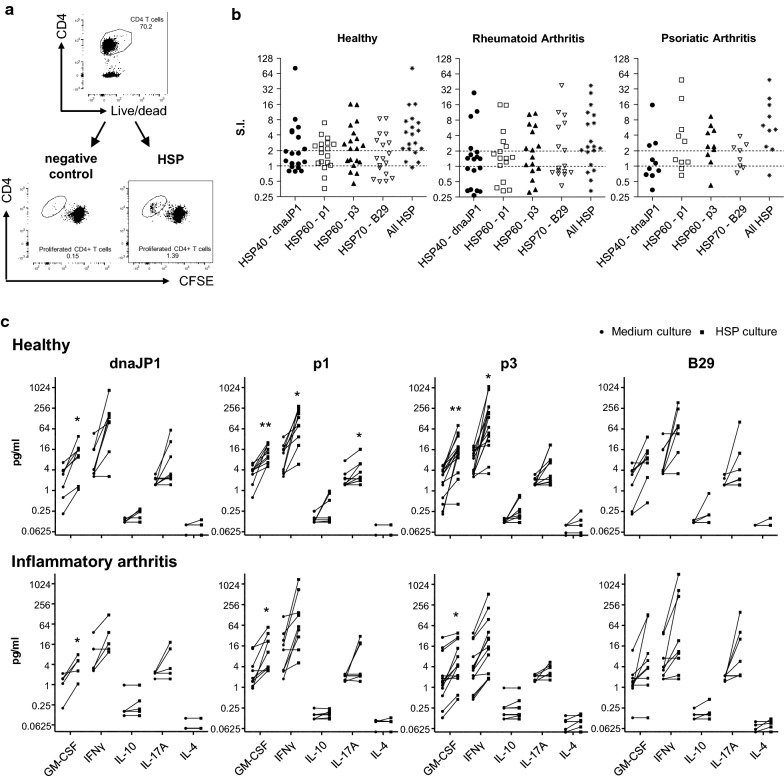
Inflammatory arthritis (IA) patients have pro-inflammatory HSP-specific CD4^+^ T-cells. Cell proliferation dye (CFSE/CTV)-labelled PBMC of healthy controls and IA patients were cultured with pan-DR-binding HSP peptides: DnaJP1, HSP60p1, HSP60p2 and B29 for 9 days. **a**, **b** Percentage of CFSE/CTV-negative live CD4^+^ T-cells was measured using flow cytometry. Gating example (**a**) and graphs with stimulation index (SI) **b** are shown. SI was measured by dividing the percentage of CFSE/CTV^−^ CD4^+^ T-cells of HSP culture by the percentage of CFSE/CTV^−^ CD4^+^ T-cells medium control (i.e. no peptide added) culture. SI > 2 was considered as increased above background proliferation. ‘All HSP’ indicates best HSP response per donor. **c** Cytokine secretion in culture supernatants was measured using MSD immunoassay. Left circle indicates cytokine concentration in medium control culture, right square indicates cytokines concentration in HSP culture. Two-tailed paired Student T-test was used. *p ≤ 0.05, **p ≤ 0.01

As shown in Fig. 1b, 78% of healthy donors, 73% of RA patients and 90% of PsA patients responded to at least one of the HSP peptides tested (Fig. 1b; see ‘All HSP’ which depicts the best HSP response for each donor). In the majority of cases, donors responded to one HSP only, but in around 10% of cases there was a detectable response to all four HSP peptides (Additional file 2: Fig S1).

To study the inflammatory nature of HSP-specific T-cell responses, we measured the secretion of the pro-inflammatory cytokines IFNγ, GM-CSF, IL-17A and IL-4 and the anti-inflammatory cytokine IL-10 in the supernatants of the HSP cultures. PBMC from both healthy donors and RA/PsA patients produced significant levels of pro-inflammatory cytokines in response to HSP (Fig. 1c).

### TolDC induce a Tr1 phenotype in antigen-specific T-cells

**Fig. 2:**
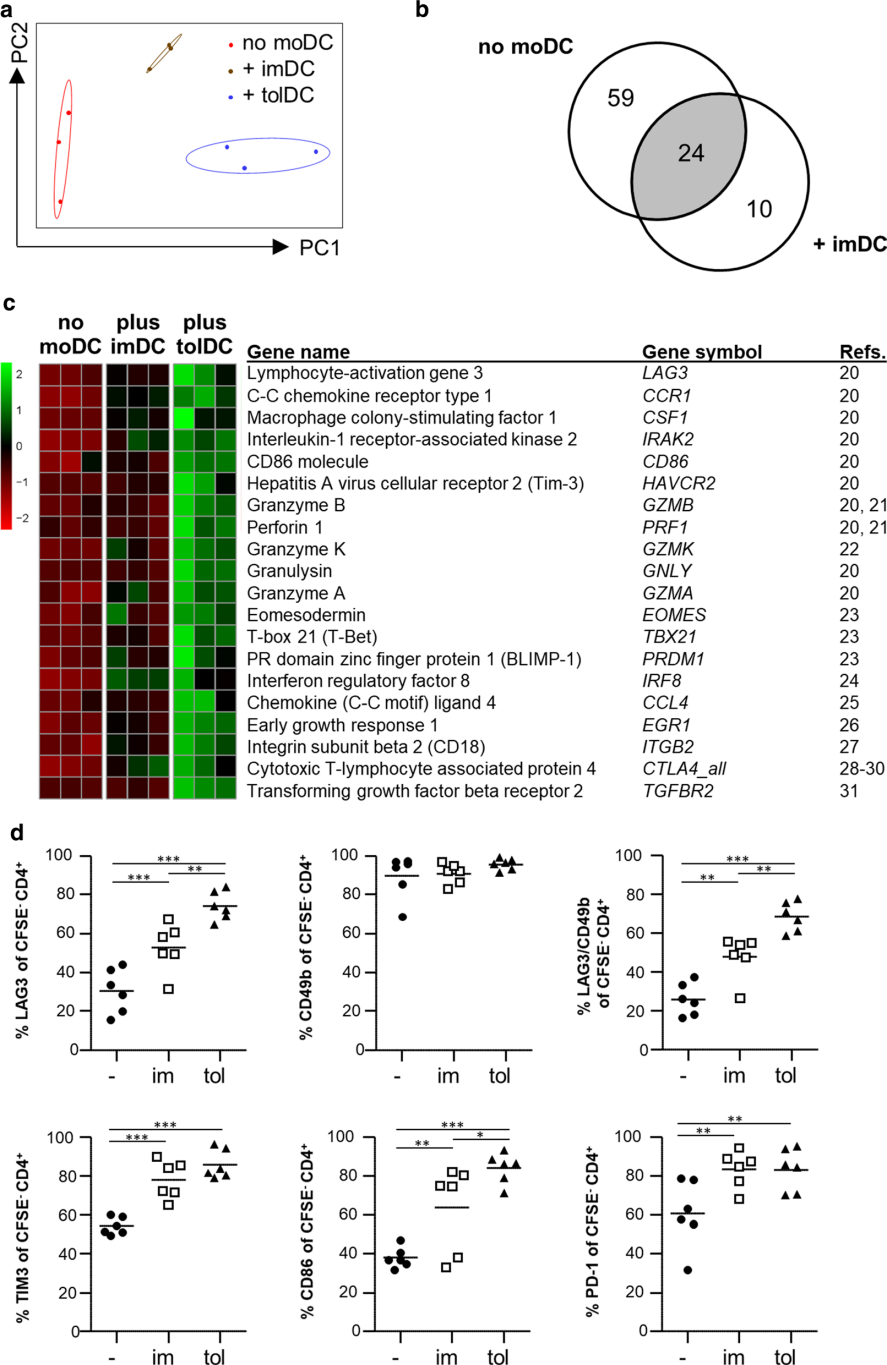
tolDC induce a Tr1 phenotype in antigen-specific CD4^+^ T cells. Cell proliferation dye (CFSE/CTV)-labelled PBMC of healthy controls were cultured with the recall antigen PPD and tolDC (tol), imDC (im) or without addition of any type of moDC (−) for 9 days. **a**–**d** 100,000 CFSE/CTV^−^ life CD4^+^ T-cells were sorted by FACS. Cell were then lysed and gene expression determined by the NanoString nCounter platform. **a** Principal component analysis (PCA) plot showing clustering of the different groups. PC1: principal component 1; PC2 principal component 2. **b** Venn diagram showing genes differentially expressed (BH P ≤ 0.1) with at least a 1.5-fold change for control (no moDC) PPD T-cells (left circle) and imDC-PPD T-cells (right circle) compared to tolDC-PPD T-cells. The numbers in the circles indicate the modulated genes for each condition. The grey area represents the overlap between both conditions. **d** Heat map of Tr1-related gene expression levels that were significantly (BH P ≤ 0.1) differentially expressed with at least a 1.5-fold change in tolDC-PPD T-cells compared to control (no moDC) PPD T-cells. **e** Percentages of LAG3, CD49b, TIM3, CD86 and PD-1 in CFSE/CTV^−^ live CD4^+^ T-cells were measured using flow cytometry. Repeated measures analysis of variance was used. *p ≤ 0.05, **p ≤ 0.01, ***p ≤ 0.001

The T-cell modulatory effects of human tolDC are usually studied in (tol)DC/T-cell co-culture models. However, because tolDC need to be able to regulate T-cell responses in the context of other immune cells, we assessed whether tolDC could regulate autologous CD4^+^ T-cells in the antigen-specific PBMC culture system described above. To this aim, we initially used the recall antigen PPD. T-cell responses to PPD were assessed in the absence or presence of tolDC. As a control, we used another DC population with known, but unstable, tolerogenic function—immature monocyte-derived DC (imDC). Mature monocyte-derived DC as a control were also considered, but we found that these mature DC induced very high background (i.e. no antigen added) proliferation of CD4, CD8 and NK cells, which was not the case when imDC or tolDC were added to the PBMC cultures (data not shown). We compared the immune-related gene-expression profile of PPD-specific T-cells activated in the absence or presence of tolDC or imDC. The gene expression profiles of the differentially activated PPD-specific T-cell groups clustered well together, indicating clear differences between treatment groups at the mRNA level (Fig. 2a). We identified 83 differentially expressed genes (DEGs) from a total of 579 genes in PPD-specific T-cells activated in the presence of tolDC (tolDC-PPD T-cells) as compared to PPD-specific T-cells activated in the absence of monocyte-derived DC (control PPD T-cells) (Benjamini–Hochberg false discovery rate 10%). Of these 83 genes, 24 DEGs were also found in tolDC-PPD T-cells as compared to PPD-specific T-cells activated in the presence of imDC (imDC-PPD T-cells). Ten additional DEGs could be identified in tolDC-PPD T-cells as compared to imDC-PPD T-cells (Fig. 2b and Additional file 3: Table S2).

Among the tolDC-PPD versus control PPD T-cell identified DEGs, 20 genes could either be identified as type 1 regulatory T-cells (Tr1)-specific genes or genes that have been described as inducers of Tr1-specific genes (Fig. 2c, [19–30]).

Recently, Gagliani et al. [19], described two surface antigens, LAG3 and CD49b, as being highly and stably expressed on Tr1 cells. Moreover, co-expression of only these two surface proteins allowed for the identification of Tr1 cells. To confirm that tolDC induce a Tr1 phenotype in PPD-specific T-cells, we measured the co-expression of LAG3 and CD49b and several other Tr1-specific proteins identified in the gene expression analysis, by flow cytometry. TolDC induced significant upregulation of the LAG3, TIM3, CD86 and PD-1 proteins as compared to control PPD cultures and the combined expression of LAG3 and CD49b could identify tolDC-induced PPD-specific Tr1 cells (Fig. 2d).

**Fig. 3:**
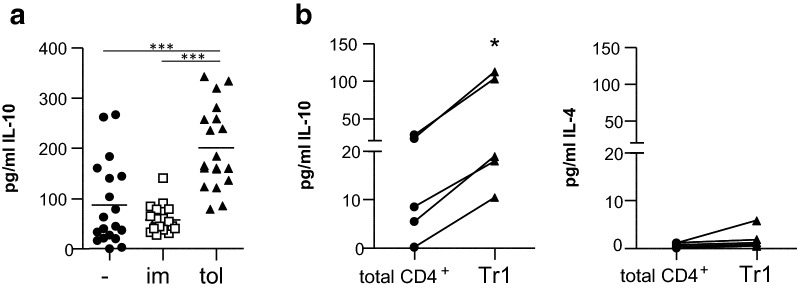
tolDC induce functional IL-10-producing antigen-specific Tr1 cells. Cell proliferation dye (CFSE/CTV)-labelled PBMC of healthy controls were cultured with the recall antigen PPD and tolDC (tol), imDC (im) or without addition of any type of moDC (−) for 9 (**a**) or 6 (**b**) days. **a** Cytokine secretion in culture supernatants was measured using ELISA. Repeated measures ANOVA was used. **b** CFSE/CTV^−^ live CD4^+^ (total CD4^+^) and CD4^+^LAG3^+^CD49b^+^ T-cells (Tr1) were sorted, rested with 10 IU/ml of IL-2 for 2 days and restimulated by plate bound anti-CD3 (10 μg/ml) and soluble anti-CD28 (1 μg/ml) for 3 days. Cytokine secretion in culture supernatants was measured using MSD immunoassay. One-tailed paired Student T-test was used. *p ≤ 0.05, **p ≤ 0.01, ***p ≤ 0.001

A hallmark of Tr1 cells is the high secretion of IL-10 in the absence of IL-4 [19, 31–33]. As shown in Fig. 3a, significantly higher levels of IL-10 were produced in the tolDC-PPD cultures as compared to the imDC-PPD or control PPD cultures. We furthermore showed that re-stimulation of sorted, proliferated LAG3^+^CD49b^+^CD4^+^ Tr1 cells produced high levels of IL-10 but no IL-4 (Fig. 3b).

Overall, these data clearly indicate that tolDC induce a Tr1 phenotype in autologous antigen-specific CD4^+^ T-cells.

### TolDC induce a Tr1 phenotype in HSP-specific T-cells of RA patients

**Fig. 4:**
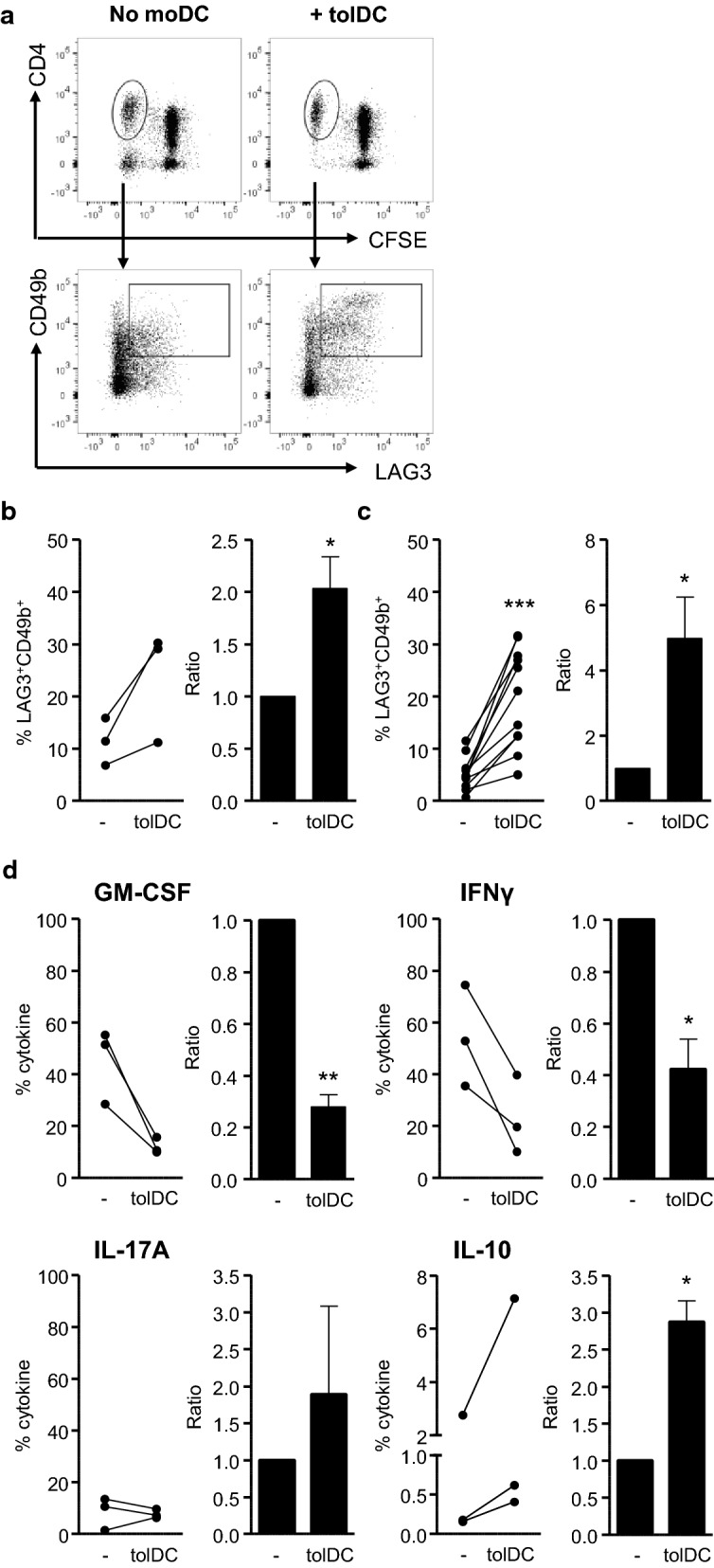
tolDC tolerise the HSP-specific T-cell response. Cell proliferation dye (CFSE/CTV)-labelled PBMC of IA patients were cultured with an HSP-peptide pool (HSP60p1, HSP60p2 and B29; 4 μg/ml per peptide) (**a, b, d**) or CA (1:1000) **(c)** and tolDC (tolDC) or without addition of any type of moDC (-) for 9 days. **a–c** Percentages and ratios of LAG3 and CD49b in CFSE/CTV^−^ life CD4^+^ T-cells were measured using flow cytometry. Gating strategy **(a)** HSP-peptide graphs (**b**) and CA graphs (**c**) are shown. Ratios were measured by dividing the percentage of CFSE/CTV^−^ CD4^+^ T-cells of tolDC cultures by the percentage of CFSE/CTV^−^ CD4^+^ T-cells of non-moDC cultures. **d** Percentages of GM-CSF, IFNγ, IL-17A and IL-10 in CFSE/CTV^−^ life CD4^+^ T-cells were measured after stimulation with PMA (50 ng/ml) ionomycin (1 μg/ml) and brefeldin A (1 μg/ml for 5 h by using flow cytometry. One-tailed (IL-10 and LAG3/CD49b) or two-tailed (all other) paired Student T-test was used. *p ≤ 0.05, **p ≤ 0.01, ***p ≤ 0.001

Since we established that HSP-specific T-cells are pro-inflammatory in both patients and healthy controls, and that the co-expression of LAG3 and CD49b can be used to identify tolDC-activated antigen-specific Tr1 cells, we used these markers to study whether tolDC induced a Tr1 phenotype in HSP-specific T-cells of IA patients. tolDC-activated HSP T-cells had significantly higher levels of LAG3/CD49b, coinciding with decreased levels of GM-CSF and IFNγ and increased IL-10 production as compared to control PPD T-cells (Fig. 4a, b and d). In addition, tolDC also induced a Tr1 phenotype in response to a third (control) antigen, *Candida albicans* (CA; Fig. 4c). Thus, tolDC are capable of inducing Tr1 responses in autologous antigen-specific CD4^+^ T-cells, irrespective of the antigen.

**Fig. 5:**
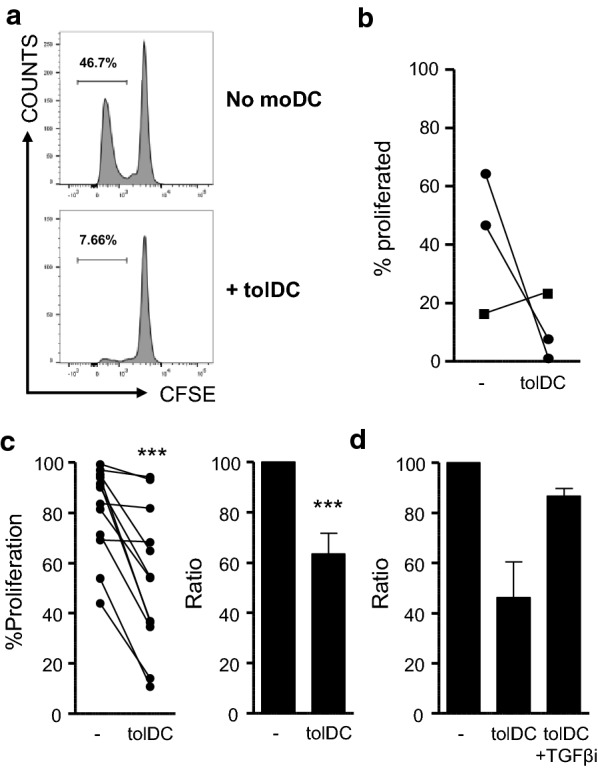
tolDC inhibit bystander NK-cell proliferation. Cell proliferation dye (CFSE/CTV)-labelled PBMC of IA patients were cultured with an HSP-peptide pool (HSP60p1, HSP60p2 and B29; 4 μg/ml per peptide) (**a, b**) or CA (1:1000) (**c, d**) and tolDC (tolDC) or without moDC (−) for 9 days. **a–d** Percentages of CFSE/CTV^−^ life NK-cells were measured using flow cytometry. Gating strategy (**a**) HSP-peptide graph **(b**) and CA graphs (**c, d**) are shown. (**d**) TGF-βRI (ALK5) inhibitor (SB-505124) was added at 1 µM. (N = 2). Ratios were measured by dividing the percentage of CFSE/CTV^−^ NK-cells of tolDC cultures by the percentage of CFSE/CTV^−^ NK-cells of non-moDC cultures. Two-tailed paired Student T-test was used. ***p ≤ 0.001

As our antigen-specific T-cell activation model using PBMC allowed us to check the behaviour of other types of immune cells, we had observed high levels of NK-cell proliferation. Interestingly, bystander NK-cell proliferation was largely inhibited by tolDC in HSP-cultures (Fig. 5a, b) and CA-cultures (Fig. 5c). We have previously shown that tolDC produce high amounts of TGFβ [17]. It is known that TGFβ can hamper NK-cell proliferation and activation [34, 35] and we therefore investigated whether blocking of the TGFβ-receptor could reverse the tolDC-induced suppression of NK-cell activation. Indeed, addition of SB-505124, a small molecule inhibitor of TGF-βRI, restored NK-cell proliferation in tolDC-CA cultures (Fig. 5d).

Altogether, our findings demonstrate that tolDC can convert pro-inflammatory antigen-specific CD4^+^ T-cells in IA (RA and PsA) patients into anti-inflammatory Tr1 cells, which are characterised by expression of LAG3/CD49b and IL-10 production. In addition, tolDC-derived TGFβ blocks bystander NK-cell activation.

## Discussion

This study focused on two key questions that require elucidation before further tolDC clinical trials in RA can commence. First, as the idea behind tolDC therapy is to dampen the autoreactive T cell response, tolDC will need ‘loading’ with an appropriate disease-relevant antigen. Second, as tolDC act in a highly targeted manner, it is necessary to have suitable biomarkers that reflect modification of antigen-specific CD4^+^ T-cell responses in order to monitor tolDC efficiency in clinical trials.

There has been debate over the last few years about the need for loading of tolDC with a disease-relevant antigen for treatment in autoimmune diseases. Several animal studies have shown that disease remission can be achieved by treatment with unpulsed tolDC [36, 37], suggesting that tolDC may be able to pick up the relevant antigens in vivo. Indeed, phase I safety trials with unloaded tolDC have been completed for diabetes and Crohn’s disease [38, 39]. The risk, however, with using non-antigen-pulsed tolDC in vivo, is that (i) it is uncertain whether tolDC pick up and present appropriate antigen(s) and (ii) the identity of the presented antigen is unknown. Thus, if it is unknown which antigens are presented by tolDC, monitoring the antigen-specific T-cells response is not possible. In addition, other animal studies have shown that loading of tolDC is crucial for their therapeutic potential. For example, we and others have shown that loading of tolDC with type II collagen was required for disease remission in the collagen-induced arthritis model [40–42]. The same is true for a mouse model of multiple sclerosis. Myelin oligodendrocyte glycoprotein-pulsed tolDC performed significantly better than unpulsed tolDC [43, 44]. We and others have therefore used autoantigen-pulsed tolDC for our phase I clinical safety trials [3, 45].

However, the search for a common antigen to load tolDC for treatment of autoimmune diseases, like RA, has been a challenge. Here, we describe the use of HSP peptides as surrogate self-antigens to load tolDC. Since HSPs are highly expressed in the inflamed tissues of patients with numerous autoimmune diseases [6–14], HSP peptides would be ideal antigens for tolDC loading, not only for the treatment of RA, but also for tolDC-based treatments of other autoimmune diseases. Because these HSP antigens are only expressed in inflamed tissues, the regulatory actions of tolDC-induced HSP-specific Tr1 cells will be targeted to the site of inflammation only. Interestingly, we found that nearly 80% of both healthy individuals and RA/PsA patients have CD4^+^ T-cell-reactivity to the HSP peptides tested, indicating the (expected) promiscuity of these peptides.

The next step was to identify how tolDC alter the antigen-specific CD4^+^ T-cell response. As with all novel therapeutic approaches, suitable biomarkers are vital for the measurement of treatment efficiency. Using several antigens, including the HSP peptides, we showed that tolDC induce a clear Tr1 phenotype in antigen-specific CD4^+^ T-cells, with high expression of the Tr1 molecules LAG3 and CD49b and the anti-inflammatory cytokine IL-10 [19, 46]. With these findings, future immune monitoring could significantly be improved by measuring the Tr1 markers LAG3/CD49b on HSP-specific—or other disease-relevant antigen-specific—MHC II tetramer^+^ CD4^+^ T-cells.

An important additional finding we report here is the striking reduction of bystander NK-cell proliferation in tolDC/PBMC co-cultures. The synovial fluid of RA patients contains high levels of NK cells and they have been shown to aggravate cytokine imbalance and inflammation in rheumatic joints [47–50]. NK cell proliferation is mainly dependent on the cytokines IL-2, IL-7 and IL-15 [51, 52] and can be hampered by factors like H_2_O_2_, soluble CD25 and TGFβ. We found that tolDC did not inhibit NK cell proliferation via IL-2, IL-15, soluble CD25 or H_2_O_2_, (data not shown), but found that NK cell proliferation could be restored by blocking TGFβ receptor signalling. This finding supports our previous notion that enhanced production of TGFβ is important for tolDC function [17] and that the tolerogenic role of tolDC in vivo might not be limited to their effect on the CD4^+^ T-cell population. Instead, tolDC could have a direct anti-inflammatory effect on the pathogenic immune cells present in the arthritic joints.

## Conclusions

tolDC induce a Tr1 phenotype in antigen-specific T-cells of both healthy individuals and IA (RA and PsA) patients. The normally pro-inflammatory HSP-specific CD4^+^ T-cells of IA patients can be converted into anti-inflammatory Tr1 cells and bystander NK-cell proliferation is hampered due to high levels of tolDC-derived TGFβ. Thus, HSP-pulsed tolDC may be a promising tool for the restoration of immune tolerance in IA patients. Indeed, we (WE and FB) are currently preparing for a phase I trial with HSP70 peptide B29-pulsed tolDC in rheumatoid arthritis patients.

## Supplementary information

**Additional file 1: Table S1.** List of reagents used for flow cytometry analysis.

**Additional file 2: Figure S1.** Inflammatory arthritis (IA) patients have CD4^+^ T-cells responding to one or more HSP-peptides. Cell proliferation dye (CFSE/CTV)-labelled PBMC of healthy controls and IA patients were cultured with pan-DR-binding HSP peptides: DnaJP1, HSP60p1, HSP60p2 and B29 for 9 days. Percentage of CFSE/CTV-negative live CD4^+^ T-cells was measured using flow cytometry. The graph depicts the percentage of donors that responds to 1, 2, 3 or all 4 HSP-peptides.

**Additional file 3: Table S2.** Differentially expressed genes in T cells co-cultured with or without tolDC or imDC.

**Additional file 4: Table S3.** Complete Nanostring dataset.

## Data Availability

All data generated or analysed during this study are included in this published article [and its additional files]. The complete processed expression data from Nanostring experiments are attached as Additional file 4: Table S3.

## Abbreviations

ANOVA: analysis of variance
CA: *Candida albicans*
DEGs: differentially expressed genes
HC: healthy controls
HSP: heat-shock proteins
IA: inflammatory arthritis
imDC: immature DC
MITAP: minimum information about tolerogenic antigen presenting cells
NK: natural killer
PBMC: peripheral blood mononuclear cells
PPD: purified protein derivative
PsA: psoriatic arthritis
RA: rheumatoid arthritis
tolDC: tolerogenic dendritic cells
Tr1: type 1 regulatory
Treg: regulatory T-cells
