# Supplementary material for: Targeting of tolerogenic dendritic cells to heat-shock proteins in inflammatory arthritis
Source: J Transl Med. 2019 Nov 14;17:375. doi: 10.1186/s12967-019-2128-4 (PMC6857208; doi:10.1186/s12967-019-2128-4)
Supplement: Supplementary file 1 — Additional file 1: Table S1. List of reagents used for flow cytometry analysis. [file 12967_2019_2128_MOESM1_ESM.docx]

**Supplementary table 1: List of reagents used for flow cytometry analysis.**

| **Marker** | **Fluorochrome** | **Clone** | **Supplier** |
| --- | --- | --- | --- |
| *Antibodies* | | | |
| CD3 | BUV395 | UCHT1 | BD Biosciences |
| CD4 | AF700 | SK3 | Biolegend |
| CD4 | APC eFluor 780 | SK3 | eBioscience |
| CD4 | BV786 | SK3 | BD Biosciences |
| CD8b | eFluor 660 | SIDI8BEE | eBioscience |
| CD19 | BV421 | HIB19 | Biolegend |
| CD56 | PE Dazzle 594 | HCD56 | Biolegend |
| CD49b | APC | P1E6-C5 | Biolegend |
| CD86 | BV711 | IT2.2 | Biolegend |
| IFNγ | AF700 | B27 | BD Biosciences |
| IL-10 | PE | JES3-19F1 | Miltenyi |
| IL-17A | APC-Cy7 | BL168 | Biolegend |
| GM-CSF | PerCP-Cy5.5 | BVD2-21C11 | Biolegend |
| LAG3 | PerCP eFluor 710 | 3DS223H | eBioscience |
| PD-1 | PE | EH12.2H7 | Biolegend |
| TIM-3 | BV650 | 7D3 | BD Biosciences |
| *Live/dead dyes* | | | |
| DAPI | - | - | Life technologies |
| Zombie aqua | - | - | Biolegend |
