# Supplementary material for: Targeting of tolerogenic dendritic cells to heat-shock proteins in inflammatory arthritis
Source: J Transl Med. 2019 Nov 14;17:375. doi: 10.1186/s12967-019-2128-4 (PMC6857208; doi:10.1186/s12967-019-2128-4)
Supplement: Supplementary file 2 — Additional file 2: Figure S1. Inflammatory arthritis (IA) patients have CD4+ T-cells responding to one or more HSP-peptides. Cell proliferation dye (CFSE/CTV)-labelled PBMC of healthy controls and IA patients were cultured with pan-DR-binding HSP peptides: DnaJP1, HSP60p1, HSP60p2 and B29 for 9 days. Percentage of CFSE/CTV-negative live CD4+ T-cells was measured using flow cytometry. The graph depicts the percentage of donors that responds to 1, 2, 3 or all 4 HSP-peptides. [file 12967_2019_2128_MOESM2_ESM.docx]

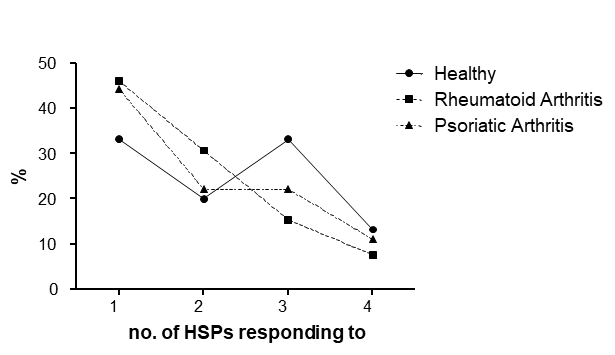


**Supplementary figure 1: Inflammatory arthritis (IA) patients have CD4^+^ T-cells responding to one or more HSP-peptides.** Cell proliferation dye (CFSE/CTV)-labelled PBMC of healthy controls and IA patients were cultured with pan-DR-binding HSP peptides: DnaJP1, HSP60p1, HSP60p2 and B29 for nine days. Percentage of CFSE/CTV-negative live CD4^+^ T-cells was measured using flow cytometry. The graph depicts the percentage of donors that responds to 1, 2, 3 or all 4 HSP-peptides.
